# Meteorological Table

**Published:** 1811-04

**Authors:** 


					[ 375 ]
METEOROLOGICAL TABLE.
From Feb. 26, to March 27.
^ I Therm, i Baifom.
?
2847 51 50 297 ? s ??*
1 44 45 42 ?6 ?7 ?
245 51 49?s ? ?
3 49 53 46?7 ?3
4 4-9 54 49 30 ?
48 53 52:297 ?5
0
47 53 47
45 49 44
292
.5
30
29 9
29 4
~4
302 ?3 ??'
|45 49 50;
51 ? 50
|49 46 43
39 45 43
43 51 46
45 52 50
49 52 49
45 50 46?3 ?
44 49 45
dry
? 5 1
? 10 8
19 10|
15
? 22 ?!
Hygrom.
17
18
19
20
121
22j
,23,
|2s
26
j46 48 44
Mil 52 45
141 54 H
? 54 521
51 54 49;
52 53 52
|51 52 53
51 52 45
|43 51 42]
44. 53 47
j44 54 46
,44 55 47
2743 53 45!?3 ? ?
?2 30
?1 30
damp
38 20
f20 ?
20 36
[22
[20 10
30 ?
35
Weather and Wind.
'23
15 3
9 ?
31 25
30 ?
? 9;C
20 19,15
11
' 5
10
6
2
- 10 -
- 15 6
5 22 13
11
6
6 24 20
10 9 4
? 5 ?
9
7 25 20
10 30 19
10 36 29
14 30 25
20 38 40
? 7 27
41
9
25
30
25
1
5
6
1
R .
R ,
F
F.
F.
C .... F.
F ... R.
C.R...
C .... R
F....?
F... ?
F
7
5
15
7
10
2
... F
... F
.... F
F...?
F
F... ?
F .... -
C.. F.
C
C..?
C.?
F .... -
F.... -
C ... F
F... ?
F
W ... S.?
.... ?.... w...
.. ? .. sw...
? f .:. ?.... w...
.. c.. w :..
... ?.... w...
... ?w ...
. c.. R .. Si.
R... W...
?sw... w..
?. SW .. NW?
NW,.N.
?.... w..
.... F.?, w..
... C .. F ... NE .
.... ? ..., NE ..
.... ? ... E ...
.... ? E
...?SE . S.E.
.... E ..
? E ..
. ? E .. .
.... W ...
R. in night.. W..
NE .. N ..
- E.. SE ..
? SE..
.... ? SE .,
? E .. *
- E ..
From the 1st to the 27th of March, a series of fair and dry weather
"38 occurred. To the 8th, indeed, the days were cloudy, and a little
rain felLin that interval On the evening of the 8th, the wind came round to the
and: ^rom thence to the E. where it has since continued, accompanied with
a cloudless sky, except on the 20th and 21st, when changing to the W. those
two days were cloudy, and a small quantity of rain fell in the night of the 21st.
The diminished humidity in the atmosphere is marked by the employment of
the dry column in the hygrometer.
The whole of March may be considered as having been remarkably tempe-
rate and genial; possessing but little of its general boisterous character: and
presenting appearances very different from those described by the poet, as cha-
racterizing this month, when
"   . Winter oft at eve resumes the breeze,
" Chills the pale morn, and bids thediiving sleets
" Deform the day delightless."
Having, io an unusual manner, been free from the " tyrannous breathings of
the
376 -
the north," pneumonic complaints have, according to our observation,
been less frequent, and less severe, than is common at this season.
Princes'Street Cavendish-Square.

				

